# Evaluating the Shelf Life and Sensory Properties of Beef Steaks from Cattle Raised on Different Grass Feeding Systems in the Western United States

**DOI:** 10.3390/foods11142141

**Published:** 2022-07-19

**Authors:** Toni L. Duarte, Bakytzhan Bolkenov, Sarah C. Klopatek, James W. Oltjen, D. Andy King, Steven D. Shackelford, Tommy L. Wheeler, Xiang Yang

**Affiliations:** 1 Department of Animal Sciences, University of California—Davis, Davis, CA 95616, USA; tlduarte@ucdavis.edu (T.L.D.); bbakytzhan@ucdavis.edu (B.B.); klopatek@ucdavis.edu (S.C.K.); jwoltjen@ucdavis.edu (J.W.O.); 2 U.S. Meat Animal Research Center, USDA-ARS, Clay Center, NE 68933, USA; andy.king@usda.gov (D.A.K.); steven.shackelford@usda.gov (S.D.S.); tommy.wheeler@usda.gov (T.L.W.)

**Keywords:** grass-fed beef, grain-fed beef, shelf life, flavor profile, palatability, tenderness

## Abstract

Consumer interest in grass-fed beef has been steadily rising due to consumer perception of its potential benefits. This interest has led to a growing demand for niche market beef, particularly in the western United States. Therefore, the objective of this study was to assess the impact of feeding systems on the change in microbial counts, color, and lipid oxidation of steaks during retail display, and on their sensory attributes. The systems included: conventional grain-fed (CON), 20 months-grass-fed (20GF), 25-months-grass-fed (25GF) and 20-months-grass-fed + 45-day-grain-fed (45GR). The results indicate that steaks in the 20GF group displayed a darker lean and fat color, and a lower oxidation state than those in the 25GF group. However, the feeding system did not have an impact on pH or objective tenderness of beef steaks. In addition, consumers and trained panelist did not detect a difference in taste or flavor between the 20GF or 25GF steaks but expressed a preference for the CON and 45GR steaks, indicating that an increased grazing period may improve the color and oxidative stability of beef, while a short supplementation with grain may improve eating quality.

## 1. Introduction

In the U.S., beef from conventional grain-fed cattle (beef that has been finished in a feedlot for over 120 days) has been the most prevalent type available to consumers [1]. However, consumer interest in grass-fed beef (beef that has been forage-fed for the duration of their lifetime), has been rapidly growing, with retail sales in the U.S. increasing from $17 million in 2012 to $480 million in 2016 [2,3]. This increased demand can be attributed to the perceived benefits that consumers believe grass-fed beef has in terms of sustainability and health [4,5,6]. Many studies have investigated the effects that grain and grass-feeding systems have on meat quality and found that grain-finished cattle tend to produce a more consistent product while meat from grass-fed animals tends to be more variable in sensory characteristics and shelf life [7,8,9,10]. For example, some studies reported that forage-fed beef had similar or greater sensory characteristics than grain-finished beef [11,12], while others reported grass-feeding to have negative impacts on quality [13,14]. Studies conducted on the consumer acceptance of grass-fed beef have resulted in mixed findings, with some consumers indicating a preference for grass-fed meat [15,16] and others indicating a preference for grain-finished meat [13,14,17].

The western region of the U.S. has seen a steadily growing demand for grass-fed beef. Situated in the western region of the U.S. is California, which has not only a growing demand for grass-fed beef, but also a substantial amount of beef cattle, with over 650,000 beef cows alone in 2017 [18]. Given its increased demand and large cattle population, it stands to reason that it is necessary to evaluate the quality and shelf life of grass-fed beef from production practices used in this region.

To date, there have been no studies that have focused specifically on beef cattle grazed in the western U.S. under feeding systems currently in practice. Additionally, there have been no studies conducted in the western U.S. investigating the effects of multiple feeding systems on meat quality and shelf life. Therefore, the objective of this study was to evaluate the shelf life and eating quality characteristics of beef steaks from animals grazed on different grass feeding systems in California and compare them to beef from the conventional grain feeding system.

## 2. Materials and Methods

Strip loins used in this study came from Angus and Angus-Hereford cross steers used in a study by Klopatek et al. [19]. The weaning, animal health protocol, and study design for that study were approved by the Institutional Animal Care and Committee at the University of California Davis (UCD; protocol #20560). After weaning, animals were randomly assigned to one of the following four feeding systems: conventional grain-finished beef (CON, *n* = 21, harvested at 18 months), 20-months-grass-fed beef (20GF, *n* = 18), 25-months-grass-fed beef (25GF, *n* = 16), and 20-month-grass-fed + 45-day-grain-finished beef (45GR, *n* = 13). All animals were grazed for 6 months on irrigated summer pasture located in Maxwell, CA. Following this initial grazing period, animals were transported to different locations based on their assigned group. Steers in the CON group were taken to the UCD feedlot (Davis, CA, USA), and transitioned onto a traditional feedlot corn-based finishing ration for 120 days and then harvested. Steers in the 20GF, 25GF and 45GR groups were shipped from Maxwell, CA to the Sierra Field Research Station in Browns Valley, CA, to graze winter-spring rangeland consisting of a mixture of grasses (*Bromus* and *Avena* spp.) and forbs (*Erodium, Medicago,* and *Trifolium* spp.). At the end of the winter-spring grazing season, 20GF animals were harvested, while steers in the 45GR group were taken to the UCD feedlot where they were transitioned onto a high-energy corn diet for a duration of 45 days before harvest. Steers in the 25GF group were brought to UCD-owned pastureland (Davis, CA, USA) that consisted of perennial grasses (*Cydon dactylon* and *Sorghum halepense*) and clover (*Medicago polymorpha* and *Trifolium dubium*), and then were harvested at 25 months of age.

### 2.1. Sample Preparation

Following harvest, strip loins (Institutional Meat Purchase Specification 180) from the right side of the carcasses were collected and vacuum-sealed under refrigerated conditions. A total of 45 strip loins (CON = 12, 20GF = 12, 45GR = 9 and 25GF = 12) were collected for the downstream analyses. The loins were transported to the UCD Meat lab (Davis, CA, USA) and wet-aged at 4–6 °C for 14 days. Following aging, the strip loins were cut from anterior to posterior into 2.54 cm steaks. Steaks were then randomly selected for either tenderness analysis (2 steaks/loin), consumer evaluation (4 steaks/loin), shelf life (3 steaks/loin), or flavor profile analysis (2 steaks/loin). Steaks for objective tenderness measurement and consumer evaluation were vacuum-packaged and stored under dark conditions at −20 °C for future analysis. Steaks for shelf life were placed on foam trays (2 W Foam Tray, CKF Inc., Toronto, Canada) with drip pads (Classic pad, Tite-Dri Industries, Boynton Beach, FL, USA). Trays were then over-wrapped using polyvinyl chloride (PVC) film (Berry AEP 1504311 18” Perforated 40 Gauge PVC Film Shrink Wrap-900/Roll, AEP Industries Inc., Montvale, NJ, USA). The steaks were moved to a commercial retail display case (Hussman, model C2NX4XLEPM; Insert Bridgeton, MO, USA) for 6 days. The display case light intensity was measured every 12 h using a light meter (Heavy Duty Datalogging Light Meter, model HD 450; Extech instruments, Nashua, NH, USA). Samples were shuffled on the shelves every 12 h to reduce the variance caused by light and temperature.

### 2.2. Instrumental Color Measurement

The objective color was measured at three positions on the lean and three positions on the external fat surface of each steak every 12 h during retail display for 6 days. Measurements were taken through the overwrap utilizing a portable spectrophotometer (Hunter MiniScan XE, model 45/O-S; Hunter Associates Laboratory Inc., Reston, VA, USA). The spectrophotometer was calibrated using black glass and white tile through PVC film before every color measurement. The Commission on Illumination (CIE) L* (lightness), a* (redness) and b* (yellowness) values were measured. The diameter of the spectrophotometer’s lens was 25 mm. In measuring external fat of the steaks, the whole diameter of the handheld spectrophotometer was covered by the thickness of the steaks.

### 2.3. Microbial Analysis

Aerobic mesophilic bacteria (AMB), aerobic psychrotrophic bacteria (APB) and lactic acid bacteria (LAB) were counted for days 0, 3 and 6 in stored steak samples. Approximately 50 g of muscle from each steak was cut into small cubes and put into a sterile Whirl-Pak filter bag (0.71 L; Nasco, Modesto, CA, USA). Then, buffered peptone water (0.1%; Difco; Becton, Dickinson and Company, Sparks, MD, USA) was added (1:2 *w*:*w*) into the same bags. Contents of the bags were homogenized using a masticator (Masticator Silver Panoramic, Neutec Group Inc, Farmingdale, NY, USA) for 2 min. The AMB and APB homogenate was serially diluted and plated on Tryptic soy agar (Difco; Becton, Dickinson and Company, Sparks, MD, USA), ehile LAB homogenate was poured and plated on MRS Agar (Difco; Becton, Dickinson and Company). The AMB and LAB plates were incubated for 48 hours at 38 °C and APB plates were incubated for 10 days at 7 °C. After incubation, all plates were counted. Microbial counts were calculated and reported as log colony forming unit (CFU)/g.

### 2.4. Measurement of pH

Around 5–10 g of samples from each steak that was used for microbial testing was mixed with 5 volumes of distilled water in a non-filtered Whirl Pack (0.71 L; Nasco, Modesto, CA, USA). Then, a pH meter (Oakton pH 700 Benchtop Meter; Cole-Parmer, Vernon Hills, IL, USA) was used to measure the pH of each sample. Prior to measurements, the pH meter was calibrated using standard solutions (pH = 7.0 and 4.0).

### 2.5. Thiobarbituric Acid Reactive Substances Analysis (TBARs)

Muscle from portions of steaks left over from day 6 microbial analysis was cut into small pieces and stored at −80 °C for subsequent TBARs analysis within a month. On the day of analysis, each stored sample was removed from the freezer, put into liquid nitrogen, and ground using a blender (Magic bullet, Capbran holdings LLC, Los Angeles, CA, USA). The resulting pulverized sample was used for TBARs analysis. Lipid oxidation was evaluated following the protocol described by Buege and Aust [20]. Results were expressed as milligrams malondialdehyde (MDA) per kg of meat.

### 2.6. Objective Tenderness Evaluation

For objective tenderness analysis, two steaks stored at −20 °C from each animal were thawed overnight and cooked on a George Foreman clamshell grill (Spectrum Brands, Middleton, WS, USA) to an internal temperature of 71 °C. The steaks were weighed prior to and following cooking to calculate the percentage of cooking loss. After cooking, one steak was immediately used for Slice Shear Force (SSF) and one was left to cool at room temperature for Warner–Bratzler Shear Force (WBSF). Using a Slice Shear Force Kit (G-R Electric Manufacturing Company LLC, Manhattan, KS, USA), 1 cm thick, 5 cm long slices were obtained. Then, the 5 cm long section was placed in the slice box with the angle of the two 45° slots lined up with the muscle fiber angle and aligned so that the slice was cut from the center of the 5 cm section. This cut provided a 1 cm thick, 5 cm long slice that was parallel to the muscle fibers. Next, the slices were placed in the testing machine (TMS Pro Texture Analyzer, Food Technology Corporation, Sterling, VA, USA) so that the blade shears perpendicular to the muscle fibers along the 5 cm dimension of the slice and samples were cut by the blade. The data was captured by the instrument software (TL-Pro software, Food Technology Corporation, Sterling, VA, USA) on the computer running the instrument.

After cooling for 240 min at room temperature, four cores were cut using WEN 8-inch 5 Speed Drill Press (WEN; Charlotte, NC, USA) from the remaining steaks parallel to the muscle fiber orientation. Cores were 1.27 cm in diameter and were sheared perpendicular to the muscle fiber using a TMS Pro Texture Analyzer (Food Technology Corporation; Sterling, VA, USA) with a Warner–Bratzler blade measuring 2.8 mm wide. The data was captured by TL-Pro software (Food Technology Corporation; Sterling, VA, USA). The setting for WBSF cross head speed was 250 mm/min and SSF crosshead speed was 500 mm/min.

### 2.7. Consumer Sensory Evaluation

Consumer sensory panel evaluations (IRB 1537841-1) were conducted at the University of California, Davis. All respondents have consented to participation in the study. One hundred and twenty untrained participants were recruited and evaluated samples over a period of six sessions. In order to be included in the current study, participants had to be between 18 and 65 years of age and had to consume beef as part of their diet. Steaks utilized for sensory evaluation were thawed at 4 °C for 24 h prior to cooking. They were then cooked to an internal temperature of 71 °C using a George Foreman clamshell grill (Spectrum Brands, Middleton, WI, USA). The internal temperature was measured at the geometric center of each steak using a K thermocouple thermometer (35100 AquaTuff, Cooper Atkins, Cincinnati, OH, USA). Following cooking, steaks were rested for 3 min and then cut into ten 1.5 cm by 1.5 cm cubes. Samples were then placed into glass bowls prelabeled with a unique 3-digit random number, covered with tin foil, and stored in an insulated food warmer (Carlisle model PC300N03, Oklahoma, OK, USA). Each participant tasted and evaluated four samples per session, meaning that each sample was evaluated by ten consumers. At the beginning of each session, participants were asked to fill out a background survey that included information about gender identity, race of origin, age, education level, household size, household income, frequency of beef consumption, grass-fed beef consumption, and most important factors influencing purchasing decisions. After filling out the background survey, participants were provided with unsalted saltine-like crackers, apple juice, and water. They were then instructed to taste their sample using the following procedure: (1) take a bite of cracker; (2) take a sip of apple juice; (3) take a sip of water; (4) smell and eat the beef sample, chewing for at least 30 s; and (5) swallow or spit out the sample. Each participant was given 2 pieces of steak cubes per sample and asked to evaluate tenderness, flavor, juiciness, and overall acceptance using a 9-point hedonic scale (1 = Dislike extremely and 9 = Like extremely).

### 2.8. Flavor Profile Analysis

Flavor analysis was conducted at the USDA-ARS U.S. Meat Animal Research Center (Clay Center, NE, USA). Per sample, 2 steaks were thawed at 5 °C for 24 h prior to cooking. They were then cooked using a conveyorized belt grill, as described by Wheeler et al. [21] for sensory panel analysis. The internal temperature of the steaks was measured at the geometric center of the steak before and following cooking using a thermocouple probe attached to a handheld thermometer (Cole-Parmer, Vernon Hills, IL, USA). Immediately following cooking, the exterior fat and connective tissue were removed, and steaks were sectioned into 1.27 cm × 1.27 cm cubes. These cubes were then mixed, randomly selected for each panelist, and immediately served. Since there was no delay between cooking and serving, all panelists evaluated samples in the same order.

A highly experienced six-member descriptive attribute panel was recruited and trained in accordance with the guidelines of Cross et al. [22] and AMSA [23]. They also received additional training in evaluating beef flavor using the lexicon, references, and definitions as described by Adhikari et al. [24]. Before initiating this study, the panelists received refresher training on the beef flavor lexicon for the specific flavor notes for this study during five one-hour sessions. Panelists rated overall tenderness and juiciness on an 8-point scale (1 = Extremely tough or dry; 8 = Extremely tender or juicy). Panelists also evaluated the flavor attributes of beef flavor identity as brown/roasted, bloody/serumy, fat-like, metallic, liver-like, green-hay-like, umami, sweet, sour, salty, bitter, barnyard, rancid, heated oil, chemical, green, asparagus, beet, buttery, spoiled/putrid, and musty/earthy/hummus on a 15-point scale (0 = Not detectable; 15 = Extremely strong). In order to avoid panel fatigue, panelists were not asked to evaluate the odor attributes described by Adhikari et al. [24] in this study. On each of five panel evaluation days, panelists were given a warm up sample from one of the treatments and eight experimental samples, two from each treatment. Only one panel session was conducted on each evaluation day. Sample order was randomized within each panel session.

### 2.9. Statistical Analysis

The experimental unit for analyses was strip loin from each animal. A completely randomized block design with repeated measures was used to analyze the L*, a*, and b* values (N = 45 × 3), pH (N = 45 × 3), and microbial data (N = 45 × 3). The two independent variables were the feeding system and retail display time (Day 0, Day 3, Day 6 for pH and microbial data; Day 0, 1, 2, 3, 4, 5, 6 for L*, a* and b* values). Therefore, a two-way ANOVA was utilized to investigate treatment effect, display time effect, and their corresponding interactive effects on color, microbial counts, and pH. Strip loin was treated as a random variable.

The TBARs values (N = 45) and the shear force values (N = 45) were analyzed using a one-way ANOVA to determine the significance of the treatments. The consumer (N = 45) data were analyzed using the Kruskal–Wallis test to determine the significance of the treatments. The Dunn’s test with *p*-value adjustment following Bonferroni methods was used for post hoc pair-wise comparisons. Flavor profile data (N = 45) were analyzed using one-way ANOVA, and treatment differences were determined using least square means with Tukey’s adjustment for *p*-value. Principal Component Analysis was conducted to analyze the relationship between feeding system and sensory flavor attributes. Data were analyzed using R statistical software (version 3.6.1; The R Foundation for Statistical Computing, Vienna, Austria). Packages ANOVA, Emmeans, Cld, and FactoMineR were used. The alpha level was defined as 0.05.

## 3. Results

### 3.1. Objective Color

**Figure 1:**
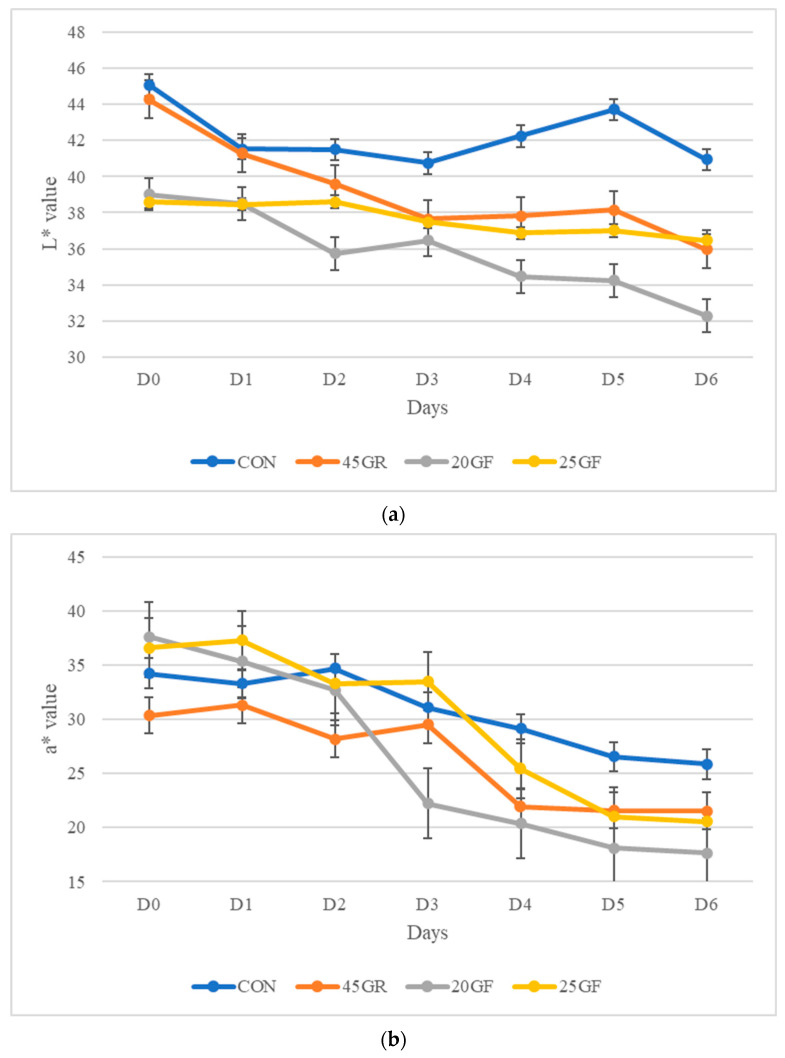

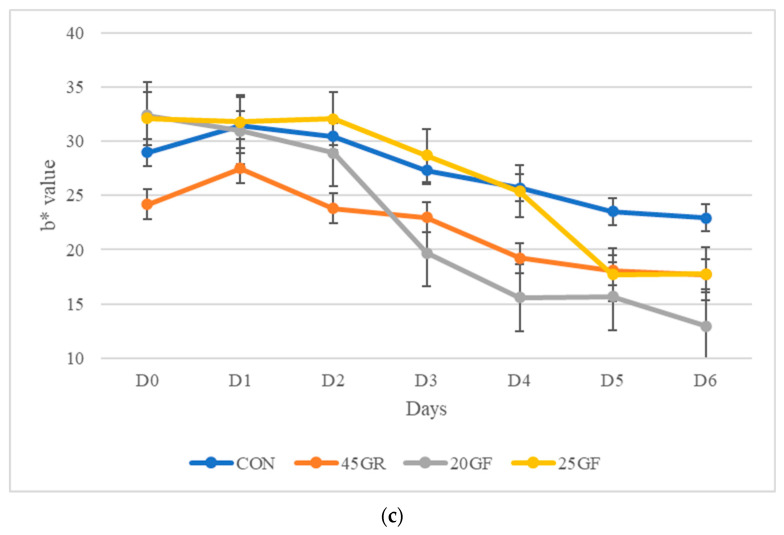
(**a**) Lean muscle L* values (lightness) of steaks over a six-day retail display period from conventional grain-finished beef (CON), 20-months-grass-fed beef (20GF), 20-months-grass-fed + 45-day-grain-finished beef (45GR), and 25-months-grass-fed beef (25GF). Steaks in the CON group possessed higher L* values throughout display time compared to the other groups (*p* < 0.05), while the 20GF group possessed the lowest L* values (*p* < 0.05). The L* values decreased for all the steaks regardless of treatment group at the end of retail display. (**b**) Lean muscle a* values (redness) of steaks over a six-day retail display period from conventional grain-finished beef (CON), 20-months-grass-fed beef (20GF), 20-months-grass-fed + 45-day-grain finished beef (45GR), and 25-months-grass-fed beef (25GF). After D2, a* values decreased dramatically for the 20GF group (*p* < 0.05) while the CON group had the highest a* values towards the end of the display period (*p* < 0.05). The a* values decreased for all the steaks regardless of treatment group at the end of retail display. (**c**) Lean muscle b* values (yellowness) of steaks over a six-day retail display period from conventional grain-finished beef (CON), 20-months-grass-fed beef (20GF), 20-months-grass-fed + 45-day-rain-finished beef (45GR), and 25-months-grass-fed beef (25GF). Treatment groups 20GF and 45 GR had the lowest b* values while the CON and 25GF groups had the highest b* values (*p* < 0.05). The b* values decreased for all the steaks regardless of treatment group at the end of retail display.

An interactive effect of feeding system and display time was detected (*p* < 0.05) for all CIE color space values of objective color of lean muscle and external fat. Steaks from the CON treatment had higher L* values in lean color than those in the 20GF or 25GF groups (*p* < 0.05; Figure 1a), but no difference in lean lightness was observed between CON and 45GR steaks until after D1 (*p* > 0.05). Similarly, following D1, 45GR steaks and 25GF steaks were similar in lightness (*p* > 0.05). However, steaks in the 20GF group were significantly (*p* < 0.05) darker than those in the 25GF group after D3. The L* values decreased for all the steaks regardless of treatment group at the end of retail display (D6). Similarly, at the end of the retail display (D5-D6), the a* values (Figure 1b) and b* (Figure 1c) values of lean muscle were significantly higher (*p* < 0.05) in the steaks from the CON than in other groups, indicating that CON group steaks were more red and yellow in color compared to other groups at the end of the display period. Following D2, there was a significant (*p* < 0.05) decline in a* values for the 20GF steaks.

**Figure 2:**
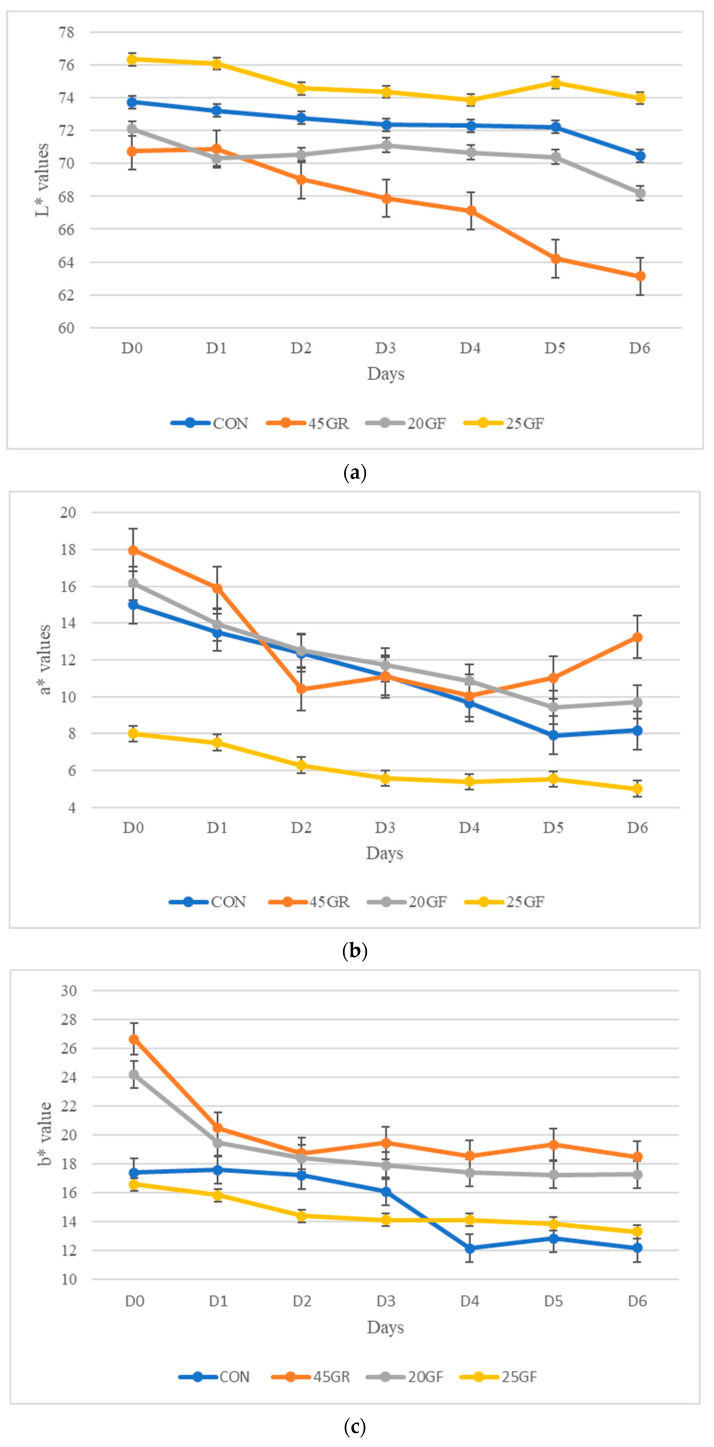
(**a**) Values of L* (lightness) for external fat of steaks over a six-day retail display period from conventional grain finished beef (CON), 20-months-grass-fed beef (20GF), 20-months-grass-fed + 45-day-grain-finished beef (45GR), and 25-months-grass-fed beef (25GF). All the groups were significantly different from each other (*p* < 0.05). Values of external fat L* were highest in 25GF followed by the CON group, while the 45GR group had the lowest values of L* for external fat. (**b**) Values of a* for external fat of steaks over a six-day retail display period from conventional grain finished beef (CON), 20-months-grass-fed beef (20GF), 20-months-grass-fed + 45-day-grain-finished beef (45GR), and 25-months-grass-fed beef (25GF). External fat a* values were lowest for 25GR throughout the retail display (*p* < 0.05). At the beginning and end of retail display, the 45GR group had the highest a* values for external fat. (**c**) Values of b* for external fat of steaks over a six-day retail display period from conventional grain finished beef (CON), 20-months-grass-fed beef (20GF), 20-months-grass-fed + 45-day-grain-finished beef (45GR), and 25-months-grass-fed beef (25GF). External fat b* values were highest in the 45GR group followed by the 20GF group (*p* < 0.05).

The lightness (L*) and redness (a*) of the external fat of steaks was significantly different among treatment groups (*p* < 0.05; Figure 2a,b). Steaks in the 25GF group had the highest L* values, while steaks in the 45GR group had the lowest L* values at later display times (after D3). Conversely, the fat of steaks in the 45GR group had the highest a* values compared to other treatment groups at the beginning of the display time (D0 and D1) and then again at the end of the display period (D5 and D6), while steaks in the 25GF group had the lowest a* values throughout the display period. All the treatment groups significantly differed from each other. Additionally, steaks in the 45GR group had the highest b* values (*p* < 0.05) followed by steaks in the 20GF group (Figure 2c). These findings indicate that the CON and 25GF groups had fat that was lighter in color while the 45GR and the 20GF groups had fat that was more yellow in color.

### 3.2. Microbial Counts

There was an interactive effect of feeding system and display time on microbial counts (*p* < 0.05; Table 1). The initial counts for LAB were higher (*p* < 0.05) in steaks in the 20GF group compared to the other treatment groups. Moreover, on day 3, AMB and APB counts for the steaks from the 20GF treatment group were above the indicative spoilage level of 7 log cfu/g [25,26,27], indicating that 20GF steaks spoiled faster than steaks from any of the other groups. On day six, counts of AMB, APB and LAB for all the treatments exceeded seven log CFU/g, which is indicative of spoilage levels [25]. Overall APB, AMB, and LAB counts were higher (*p* < 0.05) in the steaks from the 20GF treatment group while the CON group had the lowest (*p* < 0.05) bacterial counts during retail display. However, all samples were spoiled by day six, regardless of treatment group.

### 3.3. Results of pH Analysis

No significant differences were observed among treatments for pH (Table 2). Over time, pH significantly increased (*p* < 0.05) from 5.42 on day 0 to 5.68 on day 6 for the steaks in the 20GF treatment group. However, pH did not change (*p* > 0.05) by time in the other three treatment groups (data not shown).

### 3.4. TBARs Analysis Results

Results from the TBARS analysis are presented in Table 2. Steaks in the CON group possessed higher TBARS values than those in the 25GF group, with 0.79 mg MDA/kg and 0.48 mg MDA/kg, respectively (*p* < 0.05) at the end of retail display, indicating that steaks in the CON group underwent a larger degree of oxidation. In addition, steaks from the 20GF group possessed similar TBARS values to the CON group. However, all groups had TBARS values which were below the conservative unacceptable threshold of 1 mg MDA/kg [28].

### 3.5. Objective Tenderness Evaluation

No difference in objective tenderness was observed between treatments using either the slice shear force (SSF) or Warner–Bratzler shear force (WBSF) test (*p* > 0.05; Table 2). Additionally, mean WBSF values fell between 2.99 and 3.32 kgf regardless of group, indicating that they were all considered tender [29]. Likewise, the mean SSF values obtained also reflect this tenderness with all values falling between 14.74 and 16.64 kg [30].

### 3.6. Consumer Tasting Evaluation

Participants in the study were primarily female (58.8%). The majority were Asian (46.2%) and between 20 and 29 years old (64.7%; Table A1). Results from the consumer tasting evaluation are displayed in Table 3. Consumer scores for all sensory attributes had an average between 5.18–6.46 across all the treatment groups, indicating that, on average, all samples were slightly to moderately liked. However, the scores of liking of all the attributes, namely tenderness, juiciness, and flavor, were rated higher for beef steaks in the CON group when compared to both or at least one grass-fed group (20GF and 25GF; *p* < 0.05). For instance, the mean score for overall acceptance of steaks in the CON group was 6.45 compared to that of the steaks in the 20GF (5.50) and 25GF (5.51) groups. Additionally, consumers rated steaks in the CON group as having a higher overall acceptance than either the 20GF or 25GF groups (*p* < 0.05). Moreover, consumers were unable to discern any significant difference between the 45 GR and CON group for any attribute or overall acceptance (*p* > 0.05). Similarly, consumers did not detect a difference between the 20GF and 25GF group in any attribute or overall acceptance (*p* > 0.05).

### 3.7. Flavor Profile Evaluation

**Figure 3:**
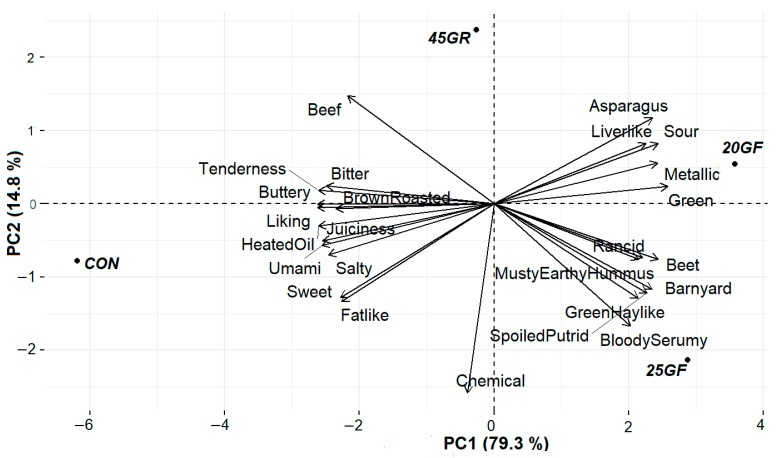
Principal Component Analysis (PCA) relating the flavor profile and consumer panel score of steaks from conventional grain finished beef (CON, *n* = 12), 20-months-grass-fed beef (20GF, *n* = 12), 20-months-grass-fed + 45-day-grain-finished beef (45GR, *n* = 9), and 25-months-grass-fed beef (25GF, *n* = 12).

The flavor profile evaluation results are presented in Table 4. Principal component analysis (PCA) of consumer acceptance and flavor profile data are illustrated in Figure 3. Principal component 1 accounts for 79.3% data variability while component 2 contributed to 14.8% data variability. These results indicate that there is a significant (*p* < 0.05) difference in the flavor profiles of beef from cattle fed under different systems. Panelists reported that positive attributes such as tenderness, fat-like, umami, sweet, salty, and buttery were more (*p* < 0.05) prevalent in grain-fed beef from the CON and 45GR groups. These attributes were also positively correlated with overall consumer liking, while negative attributes such as rancid, musty/earthy/hummus, spoiled/putrid, green hay-like, barnyard and green, were more (*p* < 0.05) associated with steaks from the 20GF and 25GF groups and were associated with a negative degree of liking by consumers. Additionally, panelists reported increased bitterness in steaks from the CON, 45GF, and 20GF treatment groups compared to the 25GF group. However, this difference was negligible, ranging from 0 to 0.14. There was no difference (*p* > 0.05) among the treatments in attributes such as juiciness, brown roasted, bloody/serumy, metallic, liverlike, heated oil, chemical, asparagus, and beet.

## 4. Discussion

### 4.1. Feeding System and Meat Color

Although meat color is not always an accurate forecaster of shelf life and safety, consumers often associate color with freshness and eating quality, and thereby discoloration of beef may lead to increased rejection, resulting in substantial economic loss [31,32,33,34]. The present study showed that beef from the 20GF group was significantly darker in color compared to all other groups (*p* < 0.05), including the 25GF group. However, steaks from the 25GF group had similar (*p* > 0.05) brightness compared to steaks from the 45GR group. Similarly to many other studies, grain-finished beef (CON) steaks had a brighter appearance compared to both the 20GF and 25GF groups. These findings agree with many other studies [35,36] that also found beef from grass-fed animals to be darker in color. This difference in brightness may be due to the effect that animal activity level has on myoglobin concentration. Studies have found that less active animals in feedlots produce meat with a brighter appearance due to the low concentrations of myoglobin compared to grass-fed animals [37]. However, additional factors such as carcass fatness, animal age, carcass weight, and intramuscular fat content may affect meat color [38].

As previously noted, steaks in the 20GF group were darker (*p* < 0.05) in color than those from all other groups, including the 25GF group, a surprising result given that many studies have shown an inverse relationship between animal age and meat lightness [39,40]. One study conducted by Bures and Barton [41] did yield similar results to the current study, finding that musculus longissimus lumborum from bulls slaughtered at 18 months was lighter than from those slaughtered at 14 months. They surmised that this difference could be due to differences in intramuscular fat content between older and younger animals. The presence of intramuscular fat can help to increase lightness due to the color of fat being lighter than that of muscle [38]. As reported by Klopatek et al. [19], steaks in the 20GF group had the lowest amount of intramuscular fat. Therefore, the difference in color observed may be due to the increased intramuscular fat in the 25GF group compared to the 20GF group, creating a lighter appearance.

In addition to increased L* values, we found that steaks in the 20GF and 45GR had lower a* values than those in the CON or 25GF groups, indicating that the latter two groups were of a more desirable red color in comparison. Generally, reports of a* values in grass-fed beef have been variable. Some studies have reported grass-fed beef to have lower a* values than grain-finished beef [42,43], while others have found grass-fed beef to have superior a* values compared to grain-finished beef [9]. Additional studies have also reported observing no difference in a* values [44,45,46]. Additionally, the difference in a* values observed between the 20GF and 25GF groups may be due to age, similar to the difference in L* values. These findings are in agreement with a previous study that showed a significant increase in a* values in Chianina beef cattle when slaughtered at 20–21 months of age rather than 18–19 months [47]. In addition to age, the accumulation of metmyoglobin also leads to meat discoloration [48]. The formation of metmyoglobin occurs naturally during retail display [48,49] but can be altered by factors such as microbial activity [50,51]. In the current study, steaks from the 20GF group had higher microbial counts than all other groups. This increased microbial activity on the surface of the 20GF steaks may reduce the oxygen level, which may have led to a decrease in redness due to the formation of metmyoglobin [52]. Similarly, the slight increase in AMB counts for steaks in the 45GR group may also have led to decreased redness. This summation is further supported by a study by Li et al. [52] which found microbial growth and meat discoloration to be closely related.

Color differences in grass-fed beef are not limited only to muscle lean meat, and are most noted in external fat color. Our results indicate that fat surface lightness was higher in the steaks from the CON and 25GF groups while fat from the 20GF and 45GR groups appeared more yellow in color. This yellowing of fat in grass-fed animals is a generally consistent finding [53,54,55,56]. The yellow color of external fat of steaks from 20GF and 45GR treatment groups might be due to the fresh pasture having increased carotenoid content [44,57]. Many studies have found meat from grass-fed animals to possess higher levels of β-carotene, the yellow/orange pigment found in plants, and reported increased yellowing of adipose tissue [58,59,60]. Following weaning, all animals in the study were grazed on irrigated pasture consisting primarily of *Cynodon dactylon* and *Sorghum halepense*. While *Sorghum halpense* is a poor source of β-carotene due to its instability during storage [61,62], Muthukrishnan et al. [63] found *Cynodon dactylon* to be a rich source of β-carotene. Although the yellowing of fat in grass-fed animals is to be expected, the yellow appearance of the fat in the 45GR steaks contradicts other studies that have found this color change to be minimized when animals were supplemented with a grain diet for as little as 28 days prior to harvest [64,65,66]. Additionally, the external fat color of the 25GF group was unexpected as it was closer to that in the CON group, meaning that it did not possess the typical yellow-colored fat expected from grass-fed animals. This lack of yellowing could be due to fluctuation in β-carotene levels due to season. A study by Barrón et al. [67] found that β-carotene levels in *Cynodon pletostachious* (African star grass) was the highest in June to August and the lowest in April. The animals in the 45GR and the 20GF groups were slaughtered between June and July. This means that they would have consumed grass with a potentially higher β-carotene level compared to the 25GF group, which was slaughtered in November. Additionally, individual variation of β-carotene metabolism in individual animals may also have affected fat color [68].

### 4.2. Feeding System and Microbial Spoilage

Most of the studies that investigated the effect of grass and grain feeding systems on shelf life evaluated color change, pH, and lipid oxidation as indicators of spoilage [9,35,69]. Other studies focused on other aspects of the effects of grass feeding have reported no significant difference in microbial counts between grain and grass-fed beef [70,71]. Our findings indicate that steaks from the 20GF group had higher microbial counts compared to all other treatments and spoiled at a faster rate. This was unexpected as we anticipated there to be no difference in microbial counts as a result of diet. There is a multitude of factors that can impact the presence and growth of spoilage microorganisms, such as processing conditions, time, temperature, and pH [72,73]. Given that previous studies mentioned had slaughtered both their grass-fed and grain fed animals in the same facility, our differences may likely be due to our animals being slaughtered at different facilities. 

Food processing plants play host to a surfeit of microorganisms that can contaminate meat at various stages of production [73,74,75]. The animals in the present study were harvested at two different facilities. In order to accurately represent current production systems, cattle were slaughtered in separate facilities based on their assigned group. Cattle in the CON and 45GR group were slaughtered at a large-scale beef facility while the 20GF and 25GF cattle were slaughtered at a smaller, natural and organic beef facility. Differences within the processing environment in these facilities may have contributed to the difference observed in the present study. Generally, the overall size of the processing facility differs between grass-fed and conventional beef. A majority of grass-fed meats are processed in small to mid-sized plants that operate at a slower pace, while conventional beef is processed in significantly larger facilities that move at a much quicker pace [76]. Therefore, it would not be unreasonable to expect inconsistent sanitation protocols may have caused an increased population of the initial spoilage bacteria on steaks from the 20GF group [77,78]. However, it is important to note that, although steaks from the 25GF system had slightly higher AMB and APB counts, they are still not significantly different (*p* > 0.05) from either the CON or 45GR steaks. Therefore, further research is needed to determine microbial differences between large-scale and small-scale plants in order to provide a clearer image more indicative of true production systems. 

### 4.3. Feeding System and Lipid Oxidation

In the present study, steaks from both the CON and the 20GF groups displayed higher TBARs values, indicating that they underwent a similar level of lipid oxidation. These results are in accord with Yang et al. [42] and Mouty et al. [79] who reported no change in lipid oxidation between grass-fed and grain-fed beef. Conversely, steaks in the 25GF group had the lowest TBARS values, which agrees with previous studies that have shown grass-fed beef to have a higher lipid oxidation stability compared to grain or mixed diets [80,81,82,83]. This increased stability is often attributed to differences in fatty acid composition. 

Many studies have reported meat from grain-fed animals to contain a higher concentration of monounsaturated fatty acids (MUFA), making them more susceptible to oxidation [84,85]. Utilizing the same groups as in this study, Klopatek et al. [86] reported a significant difference in MUFA content, with the CON group having the highest concentration. However, they also reported the 20GF group to have the lowest concentration of MUFA. This indicates that fatty acid composition may not be the primary reason for the increase in lipid oxidation seen in the 20GF group. As previously mentioned, the 25GF steaks had a higher fat content that those in the 20GF group. A previous study has shown that lean meat with very low intramuscular fat is more susceptible to lipid oxidation due to a high percentage of phospholipids [87]. This was evident in our study, where 20GF-group steaks with a low amount of intramuscular fat underwent lipid oxidation faster than 25GF- and 45 GR-group steaks. 

### 4.4. Feeding System and pH

The ultimate pH of beef can affect lean color [88]. Other studies have reported a high pH in grass-fed beef compared to grain-finished beef [89,90]. However, our measurements indicate that there was no difference in pH between the treatments. There have been previous studies that also observed an absence of pH difference in grass-fed beef together with significant color differences [83,91]. Similar to the current study, Lafreniere et al. [92] reported no difference in pH while still observing the quintessential darker appearance of grass-fed beef. As previously discussed, the color of steaks in this study followed the typical trend seen in other literature, with grass-fed beef appearing darker in color than that from animals finished on a concentrate diet. This difference in color can often be mistaken as dark, firm, and dry (DFD) beef which is associated with pre-slaughter stress. This stress results in the depletion of glycogen stores, which hinders postmortem lactic acid accumulation, resulting in insufficient pH decline [93]. Animals finished on pasture tend to have higher concentrations of myoglobin and may be more prone to pre-slaughter stress as they are not routinely handled [93,94]. However, that does not appear to be the case in this study since the pH in all groups fell between 5.51 and 5.53 (Table 3). 

### 4.5. Feeding System Effect on Tenderness, Consumer Acceptance, and Flavor Profile

There was no difference in instrumental tenderness observed among treatments in this study. This is in agreement with other studies that also reported no difference in instrumental tenderness between grass-fed and grain finished beef [95,96,97]. However, other studies have shown that tenderness is inversely related to age, with tenderness decreasing as age increases [98,99,100]. Animals in this study were slaughtered at different ages, with the CON group being the youngest at around 18 months and the 25GF group being the oldest at around 25 months of age. Therefore, we expected to see a difference in tenderness due to differences in age among the groups. Additionally, although no significant differences in instrumental tenderness were seen, consumers still rated steaks from the 20GF and 25GF groups lower than those from the CON or 45GR groups in all attributes, including tenderness. However, results from the trained sensory panel support those reported by the consumer panel, rating steaks in the 20GF and 25GF groups as less tender than those in the CON group (*p* < 0.05). These findings are consistent with other studies that have shown grass-fed beef to have lower consumer acceptability than grain finished beef among U.S. consumers [13,14]. This discrepancy between instrumental tenderness and consumer tasting evaluation is unexpected as many studies have shown a strong relationship between instrumental and consumer tenderness evaluation [101,102,103,104,105]. However, this difference between instrumental tenderness values and tenderness reported by the trained and consumer panels in this study may not be due to actual differences in tenderness, but rather differences in fat content. A study by Killinger et al. [106] found that, when presented beef steaks of similar tenderness but differing marbling levels, consumers favored steaks of a higher marbling level. Similarly, Corbin et al. [107] found that as the level of fat increased in beef steaks, so did the consumer ratings for tenderness, juiciness, flavor, and overall liking. Therefore, the increased fat content of the CON and 45GR steaks may have contributed to this perceived tenderness [108,109].

Additionally, results from this study show that both consumer and trained panels scored steaks from the 45GR group similar to CON steaks, indicating that a short period of grain supplementation may be beneficial in improving sensory attributes of grass-fed beef. This is similar to other studies that also found that supplementation of grain shortly before slaughter improved the sensory quality of meat from grass-fed animals [64,65,66]. In addition to improved sensory quality, the study by Klopatek et al. [86] that utilized the same animals as the current study found that the 45-day grain-finishing period resulted in a more desirable fatty acid profile compared to the 20GF group. 

Sensory characteristics like tenderness, juiciness, and flavor are vitally important for consumer acceptance of beef [33,102]. As mentioned previously, consumers rated steaks from the 20GF and 25GF groups lower in not only tenderness but also juiciness and flavor compared to the CON and 45GR groups. Similarly, trained panelists strongly associated negative flavor attributes with steaks from the 20GF and 25GF groups. This is consistent with other studies where panelist reported negative flavors in grass-fed beef [14,89,109]. Therefore, the results of this study may reflect that the growing demand for grass-fed beef in the U.S. may not necessarily be completely driven by sensory enjoyment of grass-fed beef but rather by consumer perception and emotion. This effect was seen in a study by Carabante et al. [17] who investigated the effect of consumer knowledge of health benefit information on consumer acceptance, emotional response, and purchase intent of grass-fed ribeye steaks. Once consumers were made aware that the sample was grass-fed and of its potential health benefits, they observed a significant increase in overall liking, purchase intent, and positive emotions. Additionally, consumer preference for grass-fed beef may differ by geographic location and cultural norms. For example, a study conducted by Realini et al. [110] found that consumers from Spain, France, and the United Kingdom preferred the taste of grass-fed beef over that of beef fed a concentrate diet. This may be due to the key difference in beef production between countries. In the U.S., grain finished beef is the predominant type available to consumers at retail. However, this is not the case in other counties, like those examined in the Realini et al. study [110], where grass-fed beef is more predominately produced and sold. Thus, consumer acceptance of grass-fed beef can be affected based on previous experience and expectations [111,112].

## 5. Conclusions

Overall, the current study found that a significant difference exists between different grass-fed systems. Animals from the 20GF system produced steaks that were darker in appearance and had a shorter microbial shelf life compared to those in the 25GF or 45GR systems. Conversely, steaks from the 25GF system displayed improved fat color and similar microbial counts to those in the CON and 45GR groups, albeit slightly higher. Additionally, the 25GF and 45GR steaks showed a lower degree of lipid oxidation compared to CON steaks. Regardless, the steaks from the 25GF and 20GF groups were rated lower in all attributes and overall acceptance by consumers compared to those in the CON and 45GR groups, a sentiment further supported by the findings of the flavor profile evaluation, where panelist associated negative flavor attributes, like rancid and spoiled, with steaks from the 20GF and 25GF groups. Additionally, consumers were unable to detect any significant differences between steaks from the 25GF group and steaks from the 20GF group. Therefore, an extended grazing period may improve the fat color and oxidative stability of grass-fed meat but does not significantly change sensory attributes. Conversely, a short period of grain supplementation, like that seen in the 45GR system, may help to improve both sensory quality and shelf-life. Therefore, further research is needed to determine the impact of forage quality and novel systems, like the 45GR system, on the sensory quality and shelf-life or grass-fed beef. These considerations must be kept in mind regarding the question of how to increase the quality of grass-fed beef in order to sustain this growing demand by meeting consumer emotional desires with sensory expectations.

## Figures and Tables

**Table 1:** Least square means of bacterial counts (log CFU/g) for beef steaks from different feeding systems^1^ during six days of retail display at 4 °C.

TYPE	DAY	CON ^1^	20GF ^1^	45GR ^1^	25GF ^1^	SEM	*p*-Value
**AMB ^2^**	D0	4.54 ^h^	5.64 ^fg^	5.05 ^gh^	4.94 ^h^	0.16	<0.01
	D3	6.11 ^ef^	7.21 ^cd^	6.61 ^de^	6.50 ^e^		
	D6	7.76 ^bc^	8.86 ^a^	8.27 ^ab^	8.16 ^b^		
**APB ^2^**	D0	4.79 ^h^	5.84 ^fg^	5.05 ^h^	5.31 ^gh^	0.17	<0.01
	D3	6.28 ^ef^	7.34 ^cd^	6.55 ^ef^	6.80 ^de^		
	D6	7.77 ^bc^	8.82 ^a^	8.03 ^bc^	8.29 ^ab^		
**LAB ^2^**	D0	4.27 ^d^	5.69 ^c^	4.62 ^d^	4.61 ^d^	0.13	<0.01
	D3	5.55 ^c^	6.97 ^b^	5.90 ^c^	5.89 ^c^		
	D6	6.77 ^b^	8.20 ^a^	7.13 ^b^	7.11 ^b^		

^1^ Treatments groups: conventional grain finished beef (CON), 20-months-grass-fed beef (20GF), 20-months-grass-fed + 45-day-grain-finished beef (45GR), and 25-months-grass-fed beef (25GF). ^2^ Aerobic mesophilic bacteria (AMB), aerobic psychrotrophic bacteria (APB), and lactic acid bacteria (LAB). ^a–h^ Least square means within rows and columns under each type of bacteria with different superscripts differed significantly (*p* < 0.05).

**Table 2:** Least square means for pH, thiobarbituric acid reactive substances (TBARS), and shear force values of steaks from cattle with different treatments stored in the retail display case at 4 °C.

	Treatments ^1^					
Traits	CON	45GR	20GF	25GF	SEM	*p*-Value
pH	5.53	5.51	5.53	5.53	0.280	0.83
TBARS	0.79 ^a^	0.60 ^bc^	0.70 ^ab^	0.48^c^	0.239	<0.01
SSF ^2^	16.64	14.74	15.79	16.57	0.456	0.56
WBSF ^3^	3.12	2.99	3.19	3.32	0.07	0.44

^1^ Treatments groups: conventional grain finished beef (CON), 20-months-grass-fed beef (20GF), 20-months-grass-fed + 45-day-grain-finished beef (45GR), and 25-months-grass-fed beef (25GF). ^2^ SSF—Slice shear force. ^3^ WBSF—Warner-Bratzler shear force. ^a–c^ Least square means within a row with different superscripts differ significantly (*p* < 0.05).

**Table 3:** Mean scores (±standard error) of four sensory attributes of steaks derived from animals under different feeding systems assessed in the consumer tasting panel evaluation ^2^.

Treatment ^1^					
Attribute	CON	20GF	45GR	25GF	*p*-Value ^3^
Tenderness	6.41 (0.18) ^a^	5.68 (0.17) ^b^	6.10 (0.16) ^ab^	5.71 (0.16) ^b^	0.001
Juiciness	5.85 (0.18) ^a^	5.52 (0.17) ^ab^	5.61 (0.18) ^ab^	5.18 (0.17) ^b^	0.049
Flavor	6.46 (0.15) ^a^	6.00 (0.17) ^b^	5.96 (0.17) ^ab^	5.62 (0.18) ^b^	0.001
Overall Acceptance	6.45 (0.16) ^a^	5.50 (0.17) ^b^	5.83 (0.18) ^ab^	5.51 (0.16) ^b^	<0.001

^1^ Treatments groups: conventional grain finished beef (CON), 20-months-grass-fed beef (20GF), 20-months-grass-fed + 45-day-grain-finished beef (45GR), and 25-months-grass-fed beef (25GF). ^2^ A 9-point hedonic scale was used for the consumer tasting panels (1 = Dislike extremely, 2 = Dislike very much, 3 = Dislike moderately, 4 = Dislike slightly, 5 = Neither like nor dislike, 6 = Like slightly, 7 = Like moderately, 8 = Like very much and 9 = Like extremely). ^3^ Kruskal–Wallis and Dunn’s test with *p*-value adjustment following Bonferroni method (pair-wise post hoc comparisons) were used to determine the significance of the treatments. The *p*-value is from the Kruskal–Wallis test. ^a,b^ Significantly different from one beef feeding group to another beef feeding group (*p* < 0.05).

**Table 4:** Flavor profile of beef steaks from different grass and grain feeding systems ^1^.

Attribute	Feeding Systems					
	CON	20GF	45GR	25GF	SEM	*p*-Value
Tenderness	6.50 ^a^	5.57 ^b^	6.09 ^ab^	5.69 ^b^	0.14	<0.01
Juiciness	5.15	4.80	4.96	4.92	0.14	0.29
Beef	4.70 ^a^	4.31 ^ab^	4.64 ^a^	4.14 ^b^	0.13	<0.01
Brown Roasted	4.01	3.67	3.89	3.81	0.22	0.66
Bloody/Serumy	0.69	0.76	0.73	0.78	0.14	0.96
Fat like	1.34 ^a^	0.69 ^b^	0.81 ^b^	0.99 ^ab^	0.13	<0.01
Metallic	1.18	0.25	0.25	0.26	0.07	0.81
Liverlike	0.15	0.37	0.42	0.40	0.09	0.11
Umami	3.16 ^a^	2.62 ^b^	2.72 ^ab^	2.68 ^b^	0.14	0.01
Sweet	0.78 ^a^	0.52	0.59 ^ab^	0.65 ^ab^	0.07	0.03
Sour	0.63	1.07	0.90	0.89	0.14	0.12
Salty	1.90 ^a^	1.47 ^b^	1.67 ^ab^	1.66 ^ab^	0.11	0.03
Bitter	0.14 ^a^	0.03 ^ab^	0.05 ^ab^	0.00 ^b^	0.03	0.04
Rancid	0.70 ^c^	1.41 ^b^	1.40 ^abc^	2.05 ^a^	0.19	<0.01
Heated Oil	0.78	0.49	0.62	0.55	0.11	0.17
Chemical	0.20	0.17	0.11	0.23	0.06	0.45
Musty/Earthy/Hummus	0.88 ^b^	1.18 ^ab^	1.20 ^ab^	1.51 ^a^	0.12	<0.01
Spoiled/Putrid	0.20 ^b^	0.50 ^ab^	0.24 ^ab^	0.52 ^a^	0.09	0.01
Buttery	1.11 ^a^	0.55 ^b^	0.76 ^ab^	0.56 ^b^	0.10	<0.01
Green Hay-like	0.47 ^b^	1.02 ^a^	0.46 ^b^	1.00 ^a^	0.12	<0.01
Barnyard	0.41 ^c^	1.24 ^ab^	0.70 ^bc^	1.56 ^a^	0.18	<0.01
Green	0.25 ^b^	0.73 ^a^	0.49 ^ab^	0.59 ^ab^	0.12	0.01
Asparagus	0.24	0.48	0.45	0.40	0.10	0.28
Beet	0.14	0.27	0.17	0.26	0.07	0.38

^1^ Feeding systems included: conventional grain finished beef (CON), 20-months-grass-fed beef (20GF), 20-months-grass-fed + 45-day-grain-finished beef (45GR), and 25-months-grass-fed beef (25GF). ^a–c^ Least square means within a row with different superscripts differ significantly (*p* < 0.05).

## Data Availability

The data presented in this study are available on request from corresponding author.

## Appendix A

**Table A1:** Demographic data of consumers (*n* = 120) that participated in tasting panel evaluation of steaks from cattle under different feeding systems ^1^.

Characteristic	Response	Percentage of Responders (%)
Gender	Male	38.66
	Female	58.82
	Other	2.52
Ethnic origin	Caucasian	21.85
	African American	0.84
	Hispanic	14.29
	Asian	46.22
	Other	16.81
Age	Under 20	14.29
	20–29	64.71
	30–39	12.61
	40–49	4.20
	50–59	2.52
	Over 60	1.68
Education Level	High school graduate	14.29
	Some college/Tech School	35.29
	College graduate	21.85
	Post graduate	28.57
Household size	1 Person	25.21
	2 People	16.81
	3 People	20.17
	4 People	20.17
	5 People	10.92
	6 People	1.68
	Over 6 People	5.04
Yearly household income	Less than $20,000	27.35
	$20,000 to $34,999	20.51
	$35,000 to $49,999	11.11
	$50,000 to $74,999	10.26
	$75,000 to $99,999	9.40
	$100,000 or more	21.37
Frequency of meat	Multiple times a day	36.44
consumption	Once a day	18.64
	Several times a week	38.14
	Once a week	5.08
	Less than once a week	1.69
Frequency of beef meat	Multiple times a day	2.56
consumption	Once a day	6.84
	Several times a week	52.99
	Once a week	29.06
	<Once a week	8.55
Frequency of grass-fed beef	At least once a week	15.25
purchase/consumption	At least once a month	25.42
	At least once every 6 months	16.95
	At least once a year	22.88
	Never	19.49
Important factors when purchasing beef	Quality	46.53
	Price	41.58
	Sustainability	3.96
	Others	6.93

**^1^** Treatments groups: CON—Conventional grain finished beef, 45GR—20 months-grass-fed + 45-day-grain-finished beef, 20GF—20-months-grass-fed beef, 25GF—25-months-grass-fed beef.
